# Developing student codesigned immersive virtual reality simulations for teaching of challenging concepts in molecular and cellular biology

**DOI:** 10.1093/femsle/fnac051

**Published:** 2022-06-07

**Authors:** F Jerry Reen, Owen Jump, Grace McEvoy, Brian P McSharry, John Morgan, David Murphy, Niall O'Leary, Billy O'Mahony, Martina Scallan, Christine Walsh, Briony Supple

**Affiliations:** School of Microbiology, University College Cork, Cork, Ireland; Synthesis and Solid State Pharmaceutical Centre, University College Cork, Cork, Ireland; Centre for the Integration of Research Teaching and Learning, University College Cork, Cork, Ireland; School of Computer Science and Information Technology, University College Cork, Cork, Ireland; School of Microbiology, University College Cork, Cork, Ireland; School of Microbiology, University College Cork, Cork, Ireland; School of Computer Science and Information Technology, University College Cork, Cork, Ireland; MAVRIC Research Lab, School of Computer Science and Information Technology, University College Cork, Cork, Ireland; School of Microbiology, University College Cork, Cork, Ireland; School of Microbiology, University College Cork, Cork, Ireland; School of Microbiology, University College Cork, Cork, Ireland; School of Computer Science and Information Technology, University College Cork, Cork, Ireland; Centre for the Integration of Research Teaching and Learning, University College Cork, Cork, Ireland

**Keywords:** virtual reality, molecular biology, cellular biology, immersive learning, virology

## Abstract

Molecular biology theory represents a critical scaffold, which underpins multiple disciplines within life sciences education. However, it is well-documented that undergraduate students can struggle to achieve deeper understanding of key concepts and/or their application. One challenging, contributory aspect is the “invisible” nature of molecular biology processes compounded by critical 3D spatial orientations of the principal components and their interactions. Molecular theory specifically requires students to construct accurate, mental spatial models to develop their understanding. However, much of the traditional teaching and examination of such theory is limited to 2D representations. Technology-enhanced, complementary teaching and examination approaches, which engage students with spatial aspects of theoretical concepts, offer an exciting opportunity to support student learning in this area. In this study, we have explored the integration of an immersive virtual reality simulation based on a challenging molecular biology concept within an existing module taught at University College Cork. A mixed methods approach, grounded in learning theory, was undertaken to assess the student user and learning experience. The consensus response from students was one of enhanced learning, understanding, engagement, and motivation. Student partnership in the process of simulation design and integration was key to delivering the fully integrated experience.

## Teaser

Student partnered design and cocreation of virtual reality simulations for teaching and learning in the molecular and cellular biology curriculum.

## Introduction

Accessing molecular and cellular biology concepts is a challenging endeavour for students (Tibell and Rundgren 2010). Molecular biology can be invisible or abstract to many students, whereby there is often no real spatial reference point in their knowledge or experience with which to conceptually appraise 2D images in lecture slides/textbooks. Furthermore, there is often significant diversity in the prior knowledge of students on courses (Hawley *et al*. 2018), making it more difficult for some students to move between content, i.e. tangible and relative to molecular abstract concepts, to abstract mathematical or computational algorithms, and so on (McComas *et al*. 2018). The skill set required to engage with molecular and cellular biology is broad, and therefore, achieving deep learning in this space requires a “holistic” approach to guiding the student. One of the emerging challenges in the education community, as set out as by Hines *et al*. (2013), is the use of technology to improve pedagogy, management, and accountability.

While the use of immersive virtual reality (I-VR) in Higher Education remains an area of active investigation, particularly with respect to accessing challenging molecular or cellular concepts (Reen *et al*. 2021), there is growing evidence of the importance of active learning in pedagogical design (Espinosa *et al*. 2020). A range of studies have explored the benefits and applications of VR in diverse educational settings, there remains a need for systematic mapping to identify design elements relevant to the application of VR in higher education (Radianti *et al*. 2020). Radianti *et al*. (2020) identified several gaps in the application of I-VR in higher education, including the lack of application of learning theory, a focus on usability rather than learning outcomes, and the fact that I-VR has typically been a part of experimental and developmental work rather than being directly applied in teaching.

Immersion, interactivity, and presence are core elements of the VR experience, itself being a culmination of the simulation (software) and headset (hardware). Previous studies have reported that students who participated in immersive learning experiences were more engaged, spent more time on the learning tasks, and acquired cognitive skills related to remembering and understanding spatial and visual information and knowledge, and better psychomotor skills such as visual or observational skills (Jensen and Konradsen 2018). The design framework underpinning the VR experience (reviewed in (Radianti *et al*. 2020)) requires extensive consideration to ensure alignment between module or curriculum learning goals and the student learning outcome. Furthermore, rather than creating a dependency between learning outcome success and the technological capabilities of any particular software/hardware combination, the design process should subordinate the technology within an appropriate, guiding pedagogical framework. The Teaching for Understanding (TfU) framework developed at Project Zero and the Harvard Graduate School of Education is of particular relevance. The framework emphasizes the role of performance approaches to making learning and understanding both visible and tangible, which underpins the context within which VR can support the student learning experience (Blythe and Perkins 1998, Wiske 1998).

In this short exploratory study, we explored the design and integration of a bespoke VR immersive experience for recombinant protein expression in an undergraduate, third year microbiology module (MB3006 “Genetic Engineering and Molecular Biotechnology” at University College Cork). The study evaluated the integration process in terms of student competency as technology users; VR immersion as a tool aligned with TfU principles of performance-based learning and; VR immersion outcomes in terms of student perspectives on learning. A mixed methods approach was taken to ensure key aspects of the student experience were captured from the broader TfU framework, while also enabling students to guide the narrative through open semistructured focus groups and interviews. Furthermore, the emergent nature of I-VR and the relative novelty it presents to students within the life science education sphere, is such that a close question survey would not likely on its own capture the student voice effectively. Hence, the need for the mixed methods approach adopted in this study. Analysis of the quantitative survey included: student perception of concept difficulty, ease of topic comprehension in microbiology, student perspective and experience in digital technologies and Teaching and Learning (T&L) modalities, digital resources, digital competencies, positive learning modalities, and access points for learning. In addition to the qualitative data, quantitative data captured in the focus groups detailed several emergent themes.

## Methods

### Participants

Students that participated in this study were recruited from those undertaking the third year Microbiology stream in March 2020. A total of 22 respondents participated in the presurvey, with three (two male and one female) students subsequently undertaking the I-VR experience. A further three students (two female and one male) were recruited for the I-VR experience from third year Microbiology in 2021, providing a robust spectrum for the data analysis. Sampling from these cohorts was random and participation was voluntary and undertaken on an anonymous basis. Finally, a student from the M.Sc. Interactive Media programme who was involved in the graphic design phase of a distinct I-VR simulation was included for a nonlife science perspective, bringing the total number to seven. It is worth noting here that Covid-19 restrictions were in force and as such sharing of hardware between students was not possible, thus limiting participation in the immersive elements of this study.

### Study design

The study presented here used a mixed methods design capturing both qualitative and quantitative data to gain insight into the emergent nature of immersive VR education within the life science education sphere. Quantitative data were captured using surveys and qualitative data were captured in semistructured focus group interviews and coded using NVivo.

### Conceptualization and realization of Immersive VR experience

I-VR design was developed initially through the M.Sc. Interactive Media degree at UCC and followed up by recruitment of a dedicated I-VR developer. A story-board approach was taken at the conceptualization phase of this study, identifying key elements of the recombinant DNA expression process that have proven challenging to teach over the years. The simulation presented students with an interactive challenge related to the construction of an expression plasmid for recombinant protein expression. Each stage of the process had knowledge and theory components, with animations providing access to the cellular context in which expression occurs. The simulations were designed to enable assessment *for* and *of* learning. Finally, the simulation was adapted for the immersive experience, being compatible with the Oculus Quest 2 headset.

### Pre-experience survey/questionnaire

A comprehensive survey was designed to (i) gauge challenges perceived by students in the teaching of various aspects of microbiology, (ii) evaluate student's experience in the virtual and digital space, and (iii) canvas views of students on how digital technologies can best be implemented in the curriculum. The survey took into consideration the criteria of Sellitz and colleagues in areas of question design such as; content/appropriateness, specific wording, permissible response formats (graded/text/multiple choice), and sequencing/ordering in the questionnaire (Sellitz *et al*. 1976). The survey was released to students through the Canvas VLE system at University College Cork, along with accompanying information relating to the study being performed. All data collected was anonymous.

### Focus groups and questionnaire

A focus group approach was purposely chosen rather than individual interviews to enable students develop their ideas and perspectives within a small group dynamic. This led students to develop insights following discussion with their peers and allowed a collective student narrative to emerge. The focus groups (five students) were semistructured based on guidelines by Krueger (2002), and were open to student driven narratives as the interviews progressed. Each was recorded (to which each participant consented) for the purpose of transcription, and this was relayed to the students prior to commencing the interviews. Due to Covid-19 safety regulations, the focus groups were run through the MS Teams platform and recorded therein. During the 1 h focus groups the facilitator guided the participants and invited each to participate in the narrative in an open and encouraging manner.

### Transcription and NVivo analysis

Transcription of the recorded focus groups was performed following the guidelines described by Udo Kuckartz (Kuckartz 2014). Initial transcription was followed by careful manual curation to ensure the fidelity of the text. Grammatical errors were not corrected in the transcription to retain the meaning and voice of the student. Subsequently, data analysis was performed using a qualitative content analysis through NVivo, applying the method of inductive category assignment. This involved manual coding based on set rules, sentiment analysis, and word cloud representation. We considered this approach based on our familiarity with the thematic area being analyzed and the extent to which the research question was defined and focused. Codes were first identified in the raw data after which organizational themes were created. An approach of (i) familiarization with the raw data, (ii) code generation, and (iii) labeling of these themes was followed. The themes and rules for inclusion during manual coding are presented in Table 1. A total of five themes were identified following this approach and these were explored further by examining direct quotes from the students as they spoke.

**Table 1.** Criteria/rules for inclusion in coding categories through NVivo.

Inductive coding category	Sample student narrative	Rules for inclusion
**Experiential learning**	*I think the VR and the acting out is kind of a similar thing, basically it is physical and real kind of learning*	Students reflect on the act of learning and the importance of an active element to that, experiencing while learning in contrast to diagrammatic learning at lectures.
**Peer learning**	*Engagement again is the main thing is you know if you're looking at a peer learning*	Students reflect on the role of peers in their learning experience.
**Presence**	*Having an actual avatar and seeing it would kind of bring you out of it*	The role of an avatar in the VR experience and the importance of feeling you are in a situation rather than observing one.
**Simulation design**	*Colour, sound, touch*	Comments and suggestions on how the VR experience can be enhanced from the student perspective.
**Student-paced learning**	*Go to a part of the VR you're interested and do that so many times until it clicks*	Students reflect on the value of being able to learn in their own space, in their own time, without the risk of “punishment.” Repetition was another criterion for inclusion here.
**Visualization**	*Sometimes you don't see it in real life, and it helps you like visualize it*	The importance of being able to visualize structures and cells, being able to see what is abstract when presented on a page.
**Virtual reality**	*I would be much more likely to want to go and use the VR than just boot up on Chrome*	Students reflected on VR itself and elements that relate directly to the technology and/or VR experience.

### Graphical and statistical analysis

GraphPad Prism was used to tabulate and graphically represent the quantitative data from the surveys. All data points were included, no exclusion criteria were applied beyond the absolute requirement for consent.

## Results and discussion

This research commenced just as Covid-19 began to impact on T&L practices, with students transitioning to online learning as physical access to the campus was interrupted. Therefore, it was an excellent opportunity for us to capture their perspective before blended or online learning became the norm. A total of 22 students undertook the Presurvey questionnaire.

### Quantitative survey-based analysis of current student learning experience and digital competency

The presurvey questions elicited a strong and consistent response with students almost unanimously disagreeing with the statements that “*Molecular constructs, such as recombinant plasmids are easy to visualize*” and “*When I experience a difficulty with course material, I approach a lecturer with questions*”. In contrast, students were in general in agreement with the statements that *“Repetition of core molecular biology concepts across modules is helpful*,” “*I think access to software that would enable me to repeat laboratory procedures virtually would increase my understanding*,” “*I think access to software that would enable me to repeat laboratory procedures virtually would enhance my ability to communicate my understanding in an MCQ exam*.” The challenges around visualization and understanding of abstract concepts such as plasmids, viruses, and cells was borne out by the survey and provided a strong basis with which to continue with the virtual design.

**Figure 1.**
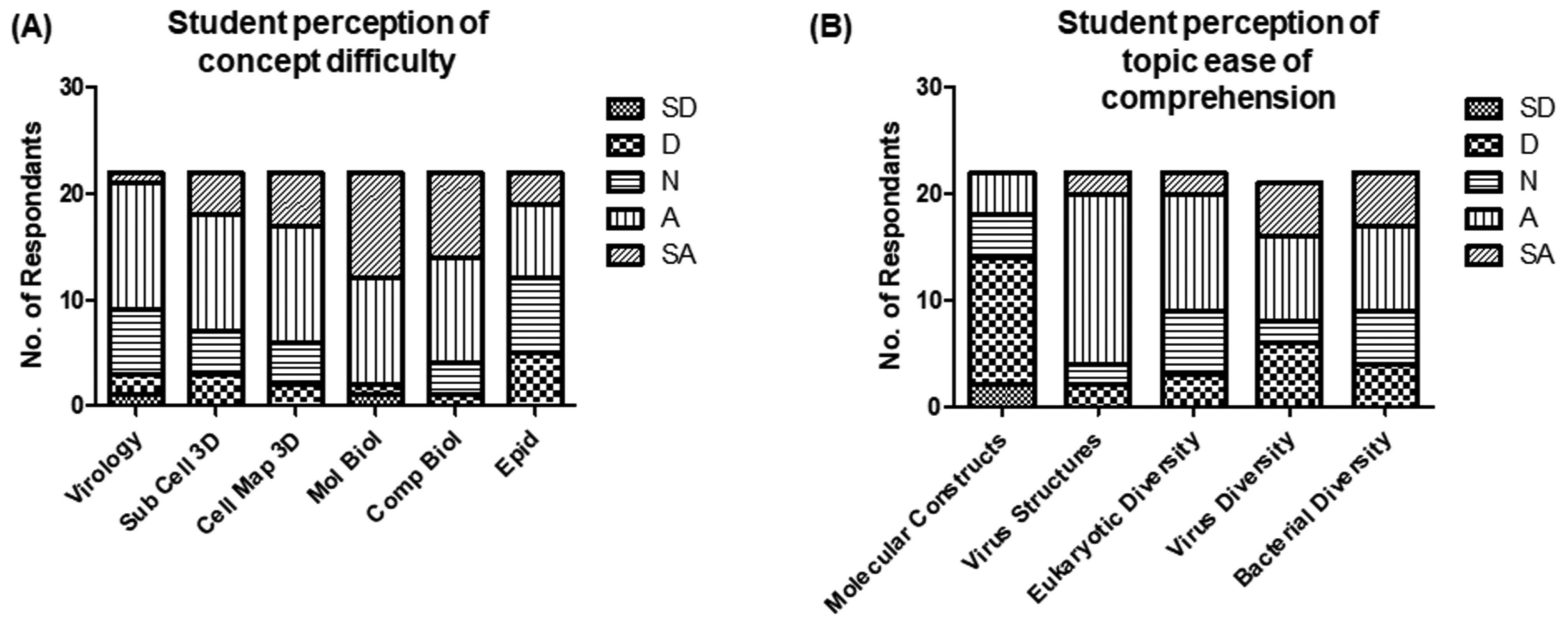
Challenging concepts in molecular and cellular biology. Students were asked their opinion on perception of **(A)** concept difficulty and **(B)** ease of topic comprehension in microbiology. A five-point scale was used to capture user responses: SD (strongly disagree), D (disagree), N (no opinion), A (agree), and SA (strongly agree). Sub Cell (Sub Cellular Structure,) Mol Biol (Molecular Biology), Comp Biol (Computational Biology), and Epid (Epidemiology).

The data also revealed further interesting patterns. First, student perception of difficulty with respect to various aspects of microbiology identified molecular biology as a significant bottleneck (Fig. 1). A total of 91% of participants either agreed or strongly agreed that molecular biology concepts are difficult to understand and grasp. In addition, when questioned about the comparative ease of comprehension of topics relating to molecular vs. microbial structure/diversity, there was a standout, negative perspective (63% of respondents) in relation to comprehension of molecular constructs.

**Figure 2.**
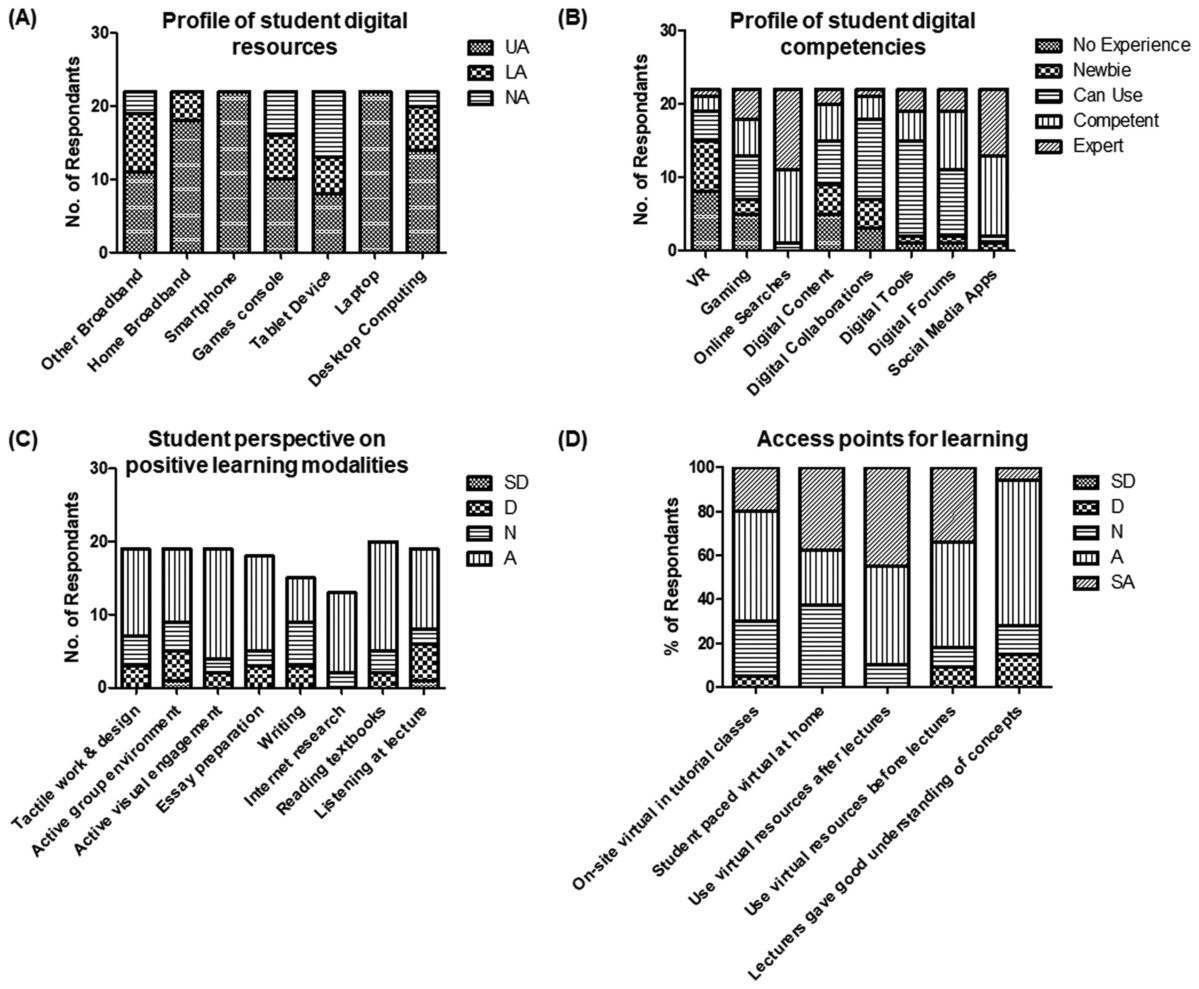
Student perspective and experience in digital technologies and T&L modalities. **(A)** Digital resources, **(B)** digital competencies, **(C)** positive learning modalities, and **(D)** access points for learning. A five-point scale was used to capture user responses: SD (strongly disagree), D (disagree), N (no opinion), A (agree), and SA (strongly agree).

Having established that molecular biology concepts are challenging for students, the next step was to ascertain their background with respect to learning with an emphasis on their digital resource competencies and access options. It was interesting to note that students by and large had access to digital technologies with the smart phone and laptop being accessible to all participants (Fig. 2A). However, while students expressed competency in various aspects of digital technology utility, only 4% and 18% considered themselves experts in virtual reality or gaming, respectively (Fig. 2B). Supports and training should, therefore, be important considerations in the design and implementation of I-VR based teaching modalities. When we explored “how students learn” it became evident that “listening in lectures” was considered the least productive form of learning (Fig. 2C). Perhaps surprisingly, “active group learning” and “writing” were the next least positively viewed modalities, though the former may be linked to a lack of experience in the student groups (Chiriac 2014). In designing the best approach to integrate virtual resources, it was clear that links to lectures were important with students expressing a desire to use virtual simulations before and after lectures where the same concepts and material were being covered (Fig. 2D).

### A qualitative study into the student narrative on I-VR

A total of seven students undertook the bespoke VR simulation fully immersive experience using the Oculus Quest 2 VR headset. While participants had previously completed the simulation on a desktop computer interface, this was the first time some of them had experienced a VR headset. Following set-up, students undertook the VR simulation at their own pace. A total of two student groups were then randomly formed into focus groups and the hour-long interviews were recorded. A total of two students provided their response in a written document. An inductive approach was taken to coding of the text (Table 1) and it revealed a strong emphasis on student paced learning, visualization, presence, and the peer–peer dynamic within the broader themes of simulation design and virtual reality. Sentiment analysis identified predominantly positive themes in the discussion of the VR intervention (Figure S1, Supporting Information), with negative comments reiterating survey responses regarding the complexity of molecular biology and the challenges/stresses around lab experiences (Parmar *et al*. 2018). The key themes are addressed in the following subsections, with the exception of simulation design which spoke to how the simulations could be developed going forward and were not intrinsically linked to the learning experience.

#### I-VR

Students reflected positively on the I-VR experience and how they could see it enhancing their learning. When probed to give an insight into how this was the case, the answers were insightful. Students found the I-VR simulation experience very accessible, straight forward, and much more interesting than anything they had experienced on a desktop computer. Students also commented on how enjoyable the experience was.

Students demonstrated a greater sense of connectivity with theoretical concepts in the immersive space where the relative spatial context of the plasmid and its components could be realized. Students felt that the natural movement afforded by the I-VR experience enhanced the learning process, consistent with the embodied cognition theory. They also commented on how the physical act of constructing the plasmid enhanced their ability to recall prior knowledge, further evidence of the constructivist through-line underpinning the I-VR experience. Indeed, several students agreed with the importance of active and physical engagement. The organization of the plasmid components made more sense to them, compared with passively observing it in a 2D diagram. One stated that:

*“When you actually physically do it yourself, if you put the parts together, it improves memory anyway to see where they go. It kind of makes you think, OK, wait, why are they where they are, or why aren't they somewhere else? So, you kind of think a bit deeper,”*

while another agreed stating that:

*“I think physically being able to pull apart a cell and putting it together is so beneficial as sometimes it is hard to visualize in your head.”*

It was interesting to note that while there was unanimous consensus regarding the positive influence of I-VR on learning and understanding, the students were unclear as to how I-VR “tricked” the brain into creating the illusion of spatial parity (Mills and Brown 2021). In essence, the quality of the simulation was sufficient to not cause students to “reject” the illusion and potentially not engage meaningfully.

#### Student paced learning

Student paced learning was something that came up in all surveys and the idea that students could visit and revisit information without penalties or judgment was a strong theme running through the student engagement. Students appreciated being able to address any aspects they did not understand over time. This was an important part of the active construction element of the experience, drawing on prior knowledge and the synthesis of ideas:

*“You would see the parts that fit and those that would not and then you would kind of go back and think OK this this applies because you know there must be this part in the plasmid because otherwise it will not work. It is kind of going back and forwards between what you knew and what you were seeing like really help me.”*

Students particularly liked the idea that they could select a specific aspect of the simulation to engage with until it “clicked” for them, and they understood it. Certain aspects may only need to be repeated once or twice whereas other elements may need to be revisited over six times before they are understood. Of course, implementation of this approach requires careful planning on the part of the tutor and the tutored, with particular care needed to scaffold the learning experience (Robinson and Persky 2020).

#### Peer–peer engagement

On a related theme, peer–peer engagement was not viewed as a requisite for learning, even though some benefits to it were recognized, particularly with respect to being able to ask for help or discuss aspects of the experience. Engaging in the I-VR simulation with friends was considered likely to act as motivation for greater engagement, though an individual approach to assessing the I-VR simulations was favored among the participants. While the importance of peer–peer engagement in VR remains to be understood, evidence from more traditional teaching modes suggests that benefit may accrue (Tullis and Goldstone 2020).

#### Presence vs. avatar

The idea of presence was a strong theme in the survey answers, with students seeing an avatar as a distraction rather than a positive influence. Several students felt that having an actual avatar and seeing it would bring them out of the experience, breaking the illusion created by I-VR. In contrast, one student could see a role for an avatar, noting that the absence of one could make the experience disorientating for the user with no spatial context within which to exist. Students did agree on a role for an avatar where the simulation related to equipment or a laboratory experience, while they also considered it important where the multiplayer function or peer engagement was in play.

#### Visualization

The visual aspect of I-VR was also important to the students. Diagrams in a textbook were viewed as 2D, and did not address meaningful concepts of scale. As a result, students often did not realize how small molecular entities are and the context within which they are located. Students who identified as visual learners felt that I-VR really enhanced the learning experience. I-VR facilitated their visualization of theory in a spatial and scale context that was only superficially represented in a traditional 2D form.

### Student partnered codesign inputs for future I-VR simulation design and implementation

**Figure 3.**
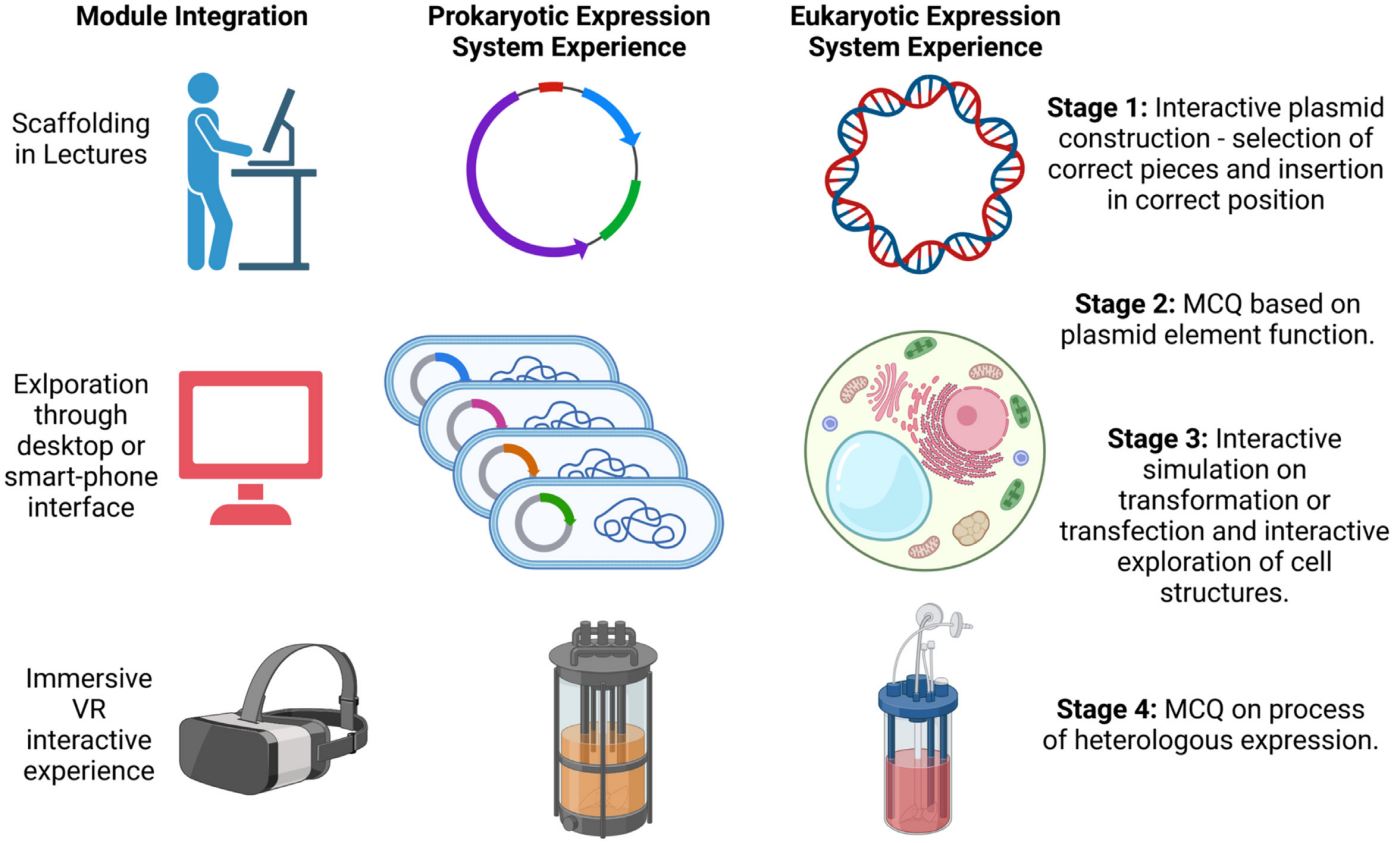
Immersive VR experiences addressing challenging molecular concepts. I-VR simulations developed to address challenges in achieving deep learning of the process of heterologous expression. Users begin by constructing a prokaryotic or eukaryotic expression plasmid, selecting the appropriate pieces, and avoiding a collection of decoys, and inserting them in the appropriate place. If successful, users then undertake an MCQ based on the function of each element inserted. Upon completion, users then proceed through an interactive simulation, arriving at the host cell which can be explored. Finally, users undertake an MCQ on the heterologous expression process, after which they can visualize and explore the protein end product. Created with BioRender.com.

In exploring the use of mixed reality platforms for student T&L, there is a need to acknowledge the advancing integration of modern life with technology and interconnectedness through the Internet of Things (IoT). Furthermore, users are at different points in engaging with technology, and questions are now arising in relation to intended use and impacts vs. user experience and outcomes in the T&L sphere. Core questions are (i) do students learn better because they are more adept at accessing information through these technologies or (ii) do students learn better because these technologies enable them to access the information in a way that opens new vistas of understanding. The positive impacts of immersion, 3D visualization and active learning present powerful drivers underpinning the utility of these technologies for deep learning in higher education (Fig. 3).

#### Spatial structure and function

Students placed high value on aspects of I-VR related to visualization, spatial awareness, a sense of structure, and exploration. In some cases, they found it difficult to explain how I-VR “tricked” the brain into a sense of presence, but there was consistency in their view that engaging with structures and getting some spatial perspective was of significant benefit in addressing concepts linked to the molecular structures. Therefore, the visuospatial dimension to I-VR is a key factor in the learning experience (Kiernan *et al*. 2021).

#### Deep learning and memory retention

Students reflected on how the plasmid made “sense” to them when they constructed the parts, they could recall the plasmid structure long after the exercise was completed. Repetition was an important feature as was the element of student-paced learning afforded to users. Similarly, effective module integration was key to achieving the enhanced learning experience, requiring bespoke module design to accommodate the I-VR content.

#### Improved physical task performance

The physical act of doing was another key feature of the study, where students commented on the construction element of the task. The active learning approach was both engaging and informative, and led students to uncover new or better understanding of the topic when compared to 2D images.

Critique of the I-VR experience was also welcomed to enable a design review that co-opted the user experience and feedback into future iterations and user supports.

#### User difficulties

The adoption of I-VR brings many challenges with respect to Universal Design for Learning (UDL) and requires careful attention to detail in the early design phase (Reen *et al*. 2021). Users can experience difficulty with the technology handling (hardware and software), interpretation of color schemes, audio aspects and the spatial nature of the structures represented. Furthermore, while the disciplinary expert might consider the simulation to be intuitive or relatively easy to navigate, the student may experience stress owing to gaps in their knowledge. Partnership with students in the I-VR design is therefore an important feature in resolving this deficit.

#### Module integration

The I-VR experience needs to be scaffolded on the module learning experience such that students can build their knowledge and understanding from the I-VR experience having sufficient prior knowledge to successfully complete all stages of the simulation. An interesting development in this space may be the tiered development of simulations where students can progress to different levels depending on their own knowledge and understanding base.

#### Learner differences

Design and implementation of the I-VR within the module must take account of the varied learning styles of students (Felten *et al*. 2013). Visual learners will likely benefit significantly from engaging with I-VR, but other more direct learners may not see the same enhancements in their own learning experience. This challenge is posed at the module level rather than specifically with the I-VR itself and should be part of the integrative design of the module from the outset.

## Conclusion

This exploratory study has provided evidence that educators can tackle particularly challenging theory for students by complementing traditional representation with immersive experiential approaches. Interdisciplinary collaboration is instrumental in delivering the intervention, as is the need for and capacity of educators to challenge anecdotal or observed student deficits with particular curriculum and actively investigate same. This has yielded positive impacts for students and provided a rich resource, which is now being reinvested into guiding T&L practice. This requires work on behalf of the educator, with efforts to develop a suitable framework for professional development ongoing in this area (Saubern *et al*. 2020). While the sample size in this study is relatively small, the outcomes of the surveys and focus groups proffered a consistent and coherent narrative that I-VR can positively enhance the learning experience of students grappling with challenging molecular and cellular concepts. Navigating that experience and its effective integration into the curriculum will require further research capturing diversity, learner styles, gender, age, geography, and other factors that will enable the design of a truly universally accessible and assessable experience.

## Authors' contributions

Conceptualization: F.J.R., O.J., B.Mc.S., J.M., D.M., N.O.L., and M.S. Funding: F.J.R., O.J., J.M., N.O.L., and M.S. Research: F.J.R. and B.O.M. performed the research and data analysis. F.J.R. wrote the first draft of this manuscript, reviewed by B.S. All authors contributed to the writing and editing of the final manuscript.

## Supplementary Material

fnac051_Supplemental_Files

## References

[bib1] Blythe T , Perkins D. The Teaching for Understanding Guide. Blythe D. (ed.), San Francisco: Jossey-Bass, 1998, 9–16.

[bib2] Chiriac EH . Group work as an incentive for learning - students' experiences of group work. Front Psychol. 2014;5:558. 24926282 10.3389/fpsyg.2014.00558 PMC4046684

[bib3] Espinosa AA , Verkade H, Mulhern TD et al. Understanding the pedagogical practices of biochemistry and molecular biology academics. Aust Educ Res. 2020;47:839–56.

[bib4] Felten P , Bagg J, Bumbry M et al. A call for expanding inclusive student engagement in SoTL. Teach Learn Inq ISSOTL J. 2013;1:63–74.

[bib5] Hawley S , Morris J, Pokrywka N et al. What are some of the major challenges in teaching/designing genetics courses today?. Trends Genet. 2018;34:82–85. 29291902 10.1016/j.tig.2017.12.003

[bib6] Hines PJ , Mervis J, McCartney M et al. Plenty of challenges for all INTRODUCTION. Science. 2013;340:291. 10.1126/science.340.6130.290 23599475

[bib7] Jensen L , Konradsen F. A review of the use of virtual reality head-mounted displays in education and training. Educ Inf Technol. 2018;23:1515–29.

[bib8] Kiernan NA , Manches A, Seery MK. The role of visuospatial thinking in students' predictions of molecular geometry. Chem Educ Res Pract. 2021;22:626–39.

[bib9] Krueger RA . Designing and Conducting Focus Group Interviews. St. Paul, MN: University of Minnesota, 2002.

[bib10] Kuckartz U . Qualitative Text Analysis. Thousand Oaks: SAGE Publications Ltd, 2014.

[bib11] McComas WF , Reiss MJ, Dempster E et al. Considering grand challenges in biology education: rationales and proposals for future investigations to guide instruction and enhance student understanding in the life sciences. Am Biol Teach. 2018;80:483–92.

[bib12] Mills KA , Brown A. Immersive virtual reality (VR) for digital media making: transmediation is key. Learn Media Technol. 2021;47:179–200.

[bib13] Parmar M , Maturi B, Dutt JM et al. Sentiment analysis on interview transcripts: an application of NLP for quantitative analysis. In: Proceedings of the 2018 International Conference on Advances in Computing, Communications and Informatics (ICACCI). p. 1063–8. Piscataway, NJ: IEEE, 2018.

[bib14] Radianti J , Majchrzak TA, Fromm J et al. A systematic review of immersive virtual reality applications for higher education: design elements, lessons learned, and research agenda. Comput Educ. 2020;147:103778.

[bib15] Reen FJ , Jump O, McSharry BP et al. The use of virtual reality in the teaching of challenging concepts in virology, cell culture and molecular biology. Front Virt Real. 2021;2:62.

[bib16] Robinson JD , Persky AM. Developing self-directed learners. Am J Pharm Educ. 2020;84:847512. 32313284 10.5688/ajpe847512 PMC7159015

[bib17] Saubern R , Urbach D, Koehler M et al. Describing increasing proficiency in teachers' knowledge of the effective use of digital technology. Comput Educ. 2020;147:103784.

[bib18] Sellitz C , Wrightsman L, Cook S. Research Methods in Social Relations. New York: Holt, Reinhart & Winston, 1976.

[bib19] Tibell LAE , Rundgren CJ. Educational challenges of molecular life science: characteristics and implications for education and research. Cbe-Life Sci Educ. 2010;9:25–33. 20194805 10.1187/cbe.08-09-0055 PMC2830159

[bib20] Tullis JG , Goldstone RL. Why does peer instruction benefit student learning?. Cogn Res. 2020;5:15. 10.1186/s41235-020-00218-5 PMC7145884 32274609

[bib21] Wiske MS. Teaching for Understanding: Linking Research with Practice. San Francisco: Jossey-Bass, 1998.

