# Supplementary material for: Developing student codesigned immersive virtual reality simulations for teaching of challenging concepts in molecular and cellular biology
Source: FEMS Microbiol Lett. 2022 Jun 7;369(1):fnac051. doi: 10.1093/femsle/fnac051 (PMC9279883; doi:10.1093/femsle/fnac051)
Supplement: fnac051_Supplemental_Files [file fnac051_supplemental_files.zip › Supplementary_Figure_S1_legends.docx]

**Supplementary Figure S1.** Manual coding of the two focus group meetings following the I-VR immersive experience with sentiment analysis performed on the complete dataset (inset). The number of references attributed to each code were as follows: virtual reality (35), simulation design (13), peer-peer (5), student-paced learning (12), presence (7), visualisation (4), and experiential learning (3). The comparatively higher % coding coverage for peer-peer reflects more detailed responses relative to other coded sections.
