# Supplementary figures and images for: Developing student codesigned immersive virtual reality simulations for teaching of challenging concepts in molecular and cellular biology
Source: FEMS Microbiol Lett. 2022 Jun 7;369(1):fnac051. doi: 10.1093/femsle/fnac051 (PMC9279883; doi:10.1093/femsle/fnac051)

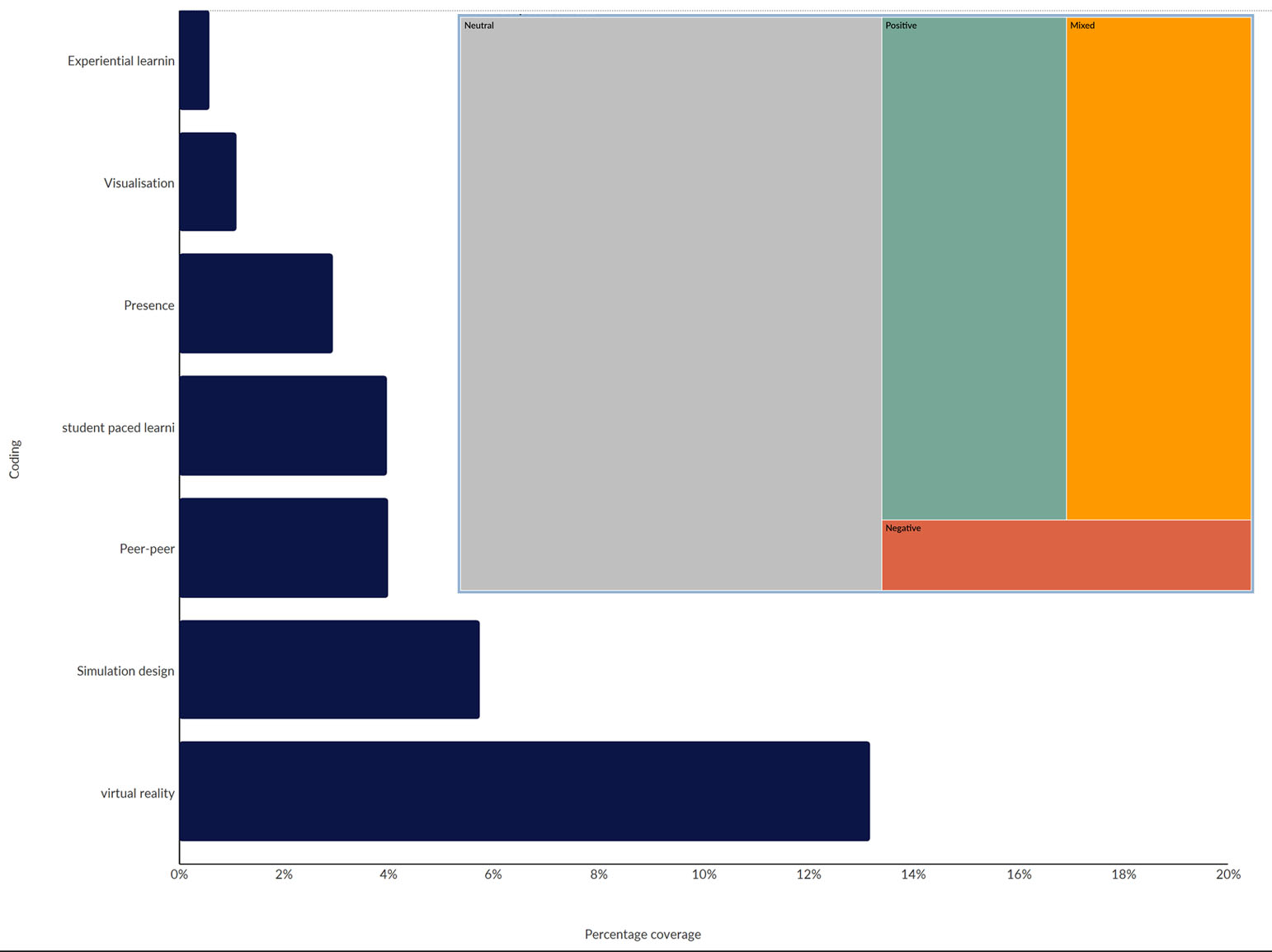

Supplement: fnac051_Supplemental_Files [file fnac051_supplemental_files.zip › Supplementary_Figure_S1.jpg]
